# Biodegradable Polymeric Substances Produced by a Marine Bacterium from a Surplus Stream of the Biodiesel Industry

**DOI:** 10.3390/bioengineering3040034

**Published:** 2016-11-30

**Authors:** Sourish Bhattacharya, Sonam Dubey, Priyanka Singh, Anupama Shrivastava, Sandhya Mishra

**Affiliations:** 1 Process Design and Engineering Cell, CSIR-Central Salt and Marine Chemicals Research Institute, Bhavnagar 364002, India; sourishb@csmcri.org; 2 Salt and Marine Chemicals, CSIR-Central Salt and Marine Chemicals Research Institute, Bhavnagar 364002, India; sonamdubey20@gmail.com; 3 DTU BIOSUSTAIN, Novo Nordisk Foundation Center for Biosustainability, Technical University of Denmark, Lyngby 2800, Denmark; prnksingh254@gmail.com; 4 Research & Product Development, Algallio Biotech Private Limited, Vadodara 390020, India; anupamashrivastav@gmail.com

**Keywords:** crude glycerol, polyhydroxyalkanoate, ε-polylysine, *Bacillus licheniformis*, fermentation

## Abstract

Crude glycerol is generated as a by-product during transesterification process and during hydrolysis of fat in the soap-manufacturing process, and poses a problem for waste management. In the present approach, an efficient process was designed for simultaneous production of 0.2 g/L extracellular ε-polylysine and 64.6% (w/w) intracellular polyhydroxyalkanoate (PHA) in the same fermentation broth (1 L shake flask) utilizing *Jatropha* biodiesel waste residues as carbon rich source by marine bacterial strain (*Bacillus licheniformis* PL26), isolated from west coast of India. The synthesized ε-polylysine and polyhydroxyalkanoate PHA by *Bacillus licheniformis* PL26 was characterized by thermogravimetric analysis (TGA), differential scanning colorimetry (DSC), Fourier transform infrared spectroscopy (FTIR), and ^1^H Nuclear magnetic resonance spectroscopy (NMR). The PHA produced by *Bacillus licheniformis* was found to be poly-3-hydroxybutyrate-co-3-hydroxyvalerate (P3HB-co-3HV). The developed process needs to be statistically optimized further for gaining still better yield of both the products in an efficient manner.

## 1. Introduction

Most of the global economy is driven by petroleum fuels as the main source of energy. However, due to market fluctuation, it is moving towards a sustainable bio based economy as fossil reserves are projected to decline completely by 2050 [1,2,3]. In addition to over exploitation of petroleum deposits, climate change and other negative environmental effects from exhaust gases lead researchers for the search of renewable alternatives such as biodiesel [4]. Biodiesel is an appealing alternative [5,6], which is clean burning, non-toxic and biodegradable [7]. It is a fatty acid methyl ester compound produced by a transesterification process of animal or plant oils with methanol in the presence of a catalyst [8,9,10]. Generally, glycerol is obtained in huge amount as a by-product in production of biodiesel [11,12]. With every 100 lbs of biodiesel produced by transesterification of vegetable oils or animal fats, 10 lbs of crude glycerol is generated [13,14]. However, the tremendous growth of the biodiesel industry has created a glycerol surplus that resulted in a dramatic 10-fold decrease in crude glycerol prices over the last few years. This decrease in prices resulted a problem for the glycerol producing and refining industries and also the economic viability of the biodiesel industry has also been greatly affected [15,16].

For sustainable development and commercialization of biodiesel production, effective utilization of crude glycerol into value added products are desired. However, conversion of crude glycerol will also promote the accretion of integrated biorefineries. 1,3-propanediol, citric acid, PHA’s, ε-polylysine, butanol, hydrogen, ethanol, phytase, lipase, succinic acid, docosahexaenoic acid, eicosapentanoic acid, monoglycerides, lipids and syngas can be produced from crude glycerol through microbial strains [17,18,19,20,21,22,23,24,25,26,27,28,29,30,31]. However, many of the available technologies need further optimization and development in the form of efficient and sustainable form for its incorporation in bio-refineries.

The utilization of low quality glycerol obtained as by-product of biodiesel production is a big challenge as this glycerol cannot be used for direct food and cosmetic uses. An effective usage for conversion of crude glycerol to specific products may cut down the biodiesel production costs. The process for biodiesel preparation and generating value added products simultaneously through the use of waste generated during the biodiesel production is a very effective approach [32]. Clearly, the development of processes to convert crude glycerol into higher-value products is an urgent need.

Various studies on tuning the material properties of the polyhydroxyalkanoate (PHA) polymer are carried out for its higher applicability in diverse areas [33]. Nerve 2010 reported production of ε-polylysine from *Streptomyces albulus* (CCRC 11814) utilizing crude glycerol as carbon source through aerobic fermentation yielding 0.2 g/L ε-polylysine. However, growth rate of *Streptomyces albulus* (CCRC 11814) was slow due to other impurities such as methanol and other salts present in crude glycerol.

ε-polylysine and polyhydroxyalkanoates are important biopolymers which can be conjugated with other biopolymers for its various applications e.g., ε-polylysine may be utilized as water absorbable hydrogels, drug carriers and anticancer agents. Simultaneously, PHA may be used in drug delivery systems, atrial septal defect repair and cardiovascular stents. ε-polylysine and polyhydroxyalkanoates are important biopolymers which are synthesized through microbe in an efficient and eco-friendly manner. However, these biopolymers may be conjugated with other biopolymers for its application as water absorbable hydrogels, drug carriers and anticancer agents. The hydrogels prepared from these complex biopolymers may be used for its application in quick peritoneal repair and prevention of post-surgical intraabdominal adhesions.

In the present study, for sustainable development of biodiesel production, efforts have been made for effective utilization of crude glycerol as the carbon source for simultaneous production of ε-polylysine and PHA. The present bioconversion route will effectively convert the waste stream of biodiesel production into value added products. In addition, metabolic engineering may used in future for improving product yield of such strains.

## 2. Materials and Methods

### 2.1. Materials

#### 2.1.1. Chemicals

Peptone, yeast extract, iron(III) citrate, NaCl, MgCl_2_, Na_2_SO_4_, CaCl_2_, KCl, NaHCO_3_, KBr, SrCl_2_, H_3_BO_3_, Na_2_O_3_Si, NaF, (NH_4_)(NO_3_) and Na_2_HPO_4_ were purchased from M/S Hi-Media Limited Mumbai, and were of the highest purity available. ε-PL was procured from Handary SA, Brussels, Belgium, Polyhydroxybutyrate and 3-hydroxyvalerate from Sigma, Bangalore, India.

#### 2.1.2. Growth and Maintenance of *Bacillus licheniformis*

*Bacillus licheniformis* was isolated from sea brine of experimental salt farm, Bhavnagar, India. It was maintained on Zobell marine agar plates containing (g/L) peptone 5.0; yeast extract 1.0; iron(III) citrate 0.1; NaCl 19.45; MgCl_2_ 8.8; Na_2_SO_4_ 3.24; CaCl_2_ 1.8; KCl 0.55; NaHCO_3_ 0.16; KBr 0.08; SrCl_2_ 0.034; H_3_BO_3_ 0.022; Na_2_O_3_Si 0.004; NaF 0.0024; (NH_4_)(NO_3_) 0.0016; Na_2_HPO_4_ 0.008; agar, 1.5, at pH 7.6 ± 0.2. 5 mL of glycerol was added to the above medium. The slants were incubated at 37 °C for 4 days and then stored at 4 °C.

Experiments were carried out in 250 mL Erlenmeyer flasks with 100 mL of production medium with following components (g/L): yeast extract, 10; glucose, 50; (NH_4_)_2_SO_4_, 15; MgSO_4_, 0.5; K_2_HPO_4_, 0.8; KH_2_PO_4_, 1.4; FeSO_4_, 0.04; ZnSO_4_, 0.04. The pH of the medium was adjusted to 6.8 with 1 N NaOH before sterilization [34]. 10% (v/v) of a 48-h-old culture (approximately 8.9 × 10^8^ cells/mL) was used as inoculum. Shake flask cultures of the organism were incubated at temperature 37 ± 2 °C with continuous agitation at 150 rpm for 96 h. These fermentation parameters were kept uniform for all the studies conducted. All experiments were carried out in triplicates.

### 2.2. Fermentation

#### 2.2.1. Culture Media

The strain *Bacillus licheniformis* PL26 was cultivated in Zobell marine broth, Himedia, Mumbai, India. The media was adjusted to pH 7.6 ± 0.2. The plates were incubated at 37 °C temperature for 48 h.

#### 2.2.2. Inoculum Development

The seed culture inoculated with loopful of *Bacillus licheniformis* was incubated overnight at 30 °C in an incubator shaker at 120 rpm. The inoculum for the production batch was prepared by using a single colony of *B. licheniformis* PL26 having 100 mL working volume.

#### 2.2.3. Simultaneous Production of ε-Polylysine and PHA

The marine bacteria was cultured in Zobell marine broth to obtain seed culture having O.D._600_ of 2.3. Zobell marine medium comprising (g/L) peptone 5.0; yeast extract 1.0; iron(III) citrate 0.1; NaCl 19.45; MgCl_2_ 8.8; Na_2_SO_4_ 3.24; CaCl_2_ 1.8; KCl 0.55; NaHCO_3_ 0.16; KBr 0.08; SrCl_2_ 0.034; H_3_BO_3_ 0.022; Na_2_O_3_Si 0.004; NaF 0.0024; (NH_4_)(NO_3_) 0.0016; Na_2_HPO_4_ 0.008 in one liter of the medium maintained at pH 7.6 ± 0.2. 20% seed culture was inoculated in the production medium which contained 20 g crude glycerol, yeast extract 5 g, (NH_4_)_2_SO_4_ 10 g, K_2_HPO_4_ 0.8 g, KH_2_PO_4_ 1.36 g, MgSO_4_ 0.5 g, ZnSO_4_ 0.04 g, FeSO_4_ 0.03 g in one litre of the medium maintained at pH 8.9 ± 0.2.

#### 2.2.4. Analysis of ε-Polylysine

The culture broth was harvested after fermentation and cells were separated by centrifugation at 15,296× *g* rcf for 10 min in refrigerated centrifuge. 1 mL of supernatant was added to 1 mL of 1 mM methyl orange, mixed thoroughly under shaking condition along with incubating it at 37 °C for 60 min [35]. Further, the solution was centrifuged at 15,296× *g* rcf for 10 min in refrigerated centrifuge and absorbance of the supernatant was measured at 465 nm on UV-vis spectrophotometer (Varian, Palo Alto, CA, USA). A standard curve was derived from measurements with known amounts (0.1–2 mg/mL) of standard ε-PL procured from Handary S.A. [36].

#### 2.2.5. Percentage Carbon Utilization of *Bacillus licheniformis* PL26

Percentage carbon utilized by *Bacillus licheniformis* PL26 was calculated a

(1) $$\mathrm{Carbon}\;\mathrm{utilization}\;\left(\%\right)\;=\;\frac{\mathrm{Total}\;\mathrm{utilized}\;\mathrm{carbon}\;\mathrm{by}\;\mathrm{bacteria}}{\mathrm{Total}\;\mathrm{carbon}\;\mathrm{present}\;\mathrm{in}\;\mathrm{the}\;\mathrm{medium}}\;\times 100$$

Glycerol estimation was carried out by using “Waters Alliance” high performance chromatographic system equipped with RI detector (Waters 2414 model, Waters India Ltd., Bangalore, India) and separation module (Waters 2695 model, Waters India Ltd., Bangalore, India). Chromatographic separations were performed on an “Aminex HPX-87H” column (300 × 7.8 mm) (Bio-Rad Laboratories, Richmond, CA, USA) with a precolumn (30 × 4.6 mm) of the same stationary phase (DVB-S, hydrogen form, Richmond, CA, USA). Isocratic elution at a flow rate of 0.6 mL/min was carried out using a mixture of 5 mM sulfuric acid. Peak detection was made by keeping the cells of the RI detector at 30 °C. The samples were appropriately degassed, twice diluted with double-distilled water, filtered through a “Whatman” 0.45-μm filter membrane (GE Healthcare Life Sciences, Little Chalfont, Buckinghamshire, UK), and then injected (50-μL loop volume). Data were obtained and processed by using “Waters EMPOWER” software (waters Corporation India, Bangalore, India). Peak identification was carried out by spiking the sample with pure standards and comparing the retention times with those of pure compounds.

#### 2.2.6. Extraction and Purification of PHA

After completion of 96 h fermentation, culture broth was centrifuged at 15,296× *g* rcf for 10 min in refrigerated centrifuge. The cell pellets were oven dried overnight at 60 °C. Cellular digestion of dried cell pellet was carried out by re-suspending it in 6% (v/v) sodium hypochlorite solution followed by centrifugation at 10,000 rpm for 5 min. Further, the digested cell pellets were washed twice with methanol followed by distilled water to remove the traces of impurities resulting in a purified product, which was further dissolved in chloroform and weighed after air drying [37].

### 2.3. Purification of *ε*-Polylysine

#### 2.3.1. Precipitation of Polycationic ε-PL with TPB^−^ Anion from the Supernatant

After removal of cells through centrifugation, the supernatant obtained was treated with sodium tetraphenylborate for precipitating ε-PL as a polyelectrolyte salt with the TPB^−^ anion. The polycationic ε-PL salt with the TPB^−^ anion was further purified by washing the mixed precipitate with acetone to remove triphenylborate and benzene. Thereafter, the precipitate reacted with 1 M HCl for obtaining ε-PL hydrochloride.

#### 2.3.2. Analytical Methods

FT-IR spectra of obtained PHA were recorded on a Perkin-Elmer Spectrum GX (FT-IR System, Waltham, MA, USA) instrument. ^1^H Nuclear magnetic resonance spectroscopy of PHA was determined on Bruker Avance-II 500 (Ultra shield) spectrometer, Bangalore, India, at 500 MHz, in CDCl_3_. Proton ^1^H NMR spectroscopy was also used to determine copolymer composition through running standards of 3HB and 3HV. Differential scanning calorimetry (DSC) of PHA was carried out using a DSC 204 F1 phoenix instrument with Netzsch software (NETZSCH Technologies India Pvt. Ltd., Chennai, India). The PHA samples were scanned from −20 °C to 500 °C with the heating rate of 10 °C/min. Glass transition temperature and onset melting points were determined in the scan between −20 °C and 500 °C in DSC analysis. Thermo-gravimetric analysis (TGA) of PHA was carried out in temperature range of 27–500 °C using TG 209 F1 instrument (NETZSCH Technologies India Pvt. Ltd., Chennai, India).

## 3. Results and Discussions

### 3.1. Simultaneous *ε*-Polylysine and PHA Production by B. licheniformis PL26

After complete submerged fermentation of 96 h at an agitation of 220 rpm, fermentation broth was centrifuged to obtain supernatant containing ε-polylysine and biomass for PHA extraction.

Simultaneous production of ε-polylysine and PHA by *B. licheniformis* PL26 was obtained utilizing crude glycerol as the carbon source. However, *B. licheniformis* is able to produce 0.2 g/L ε-polylysine extracellularly in the fermentation broth along with 64.59% PHA with respect to dry cell weight i.e., 1.1 g/L P(3HB-co-3HV) having 96 h production age at 37 °C (Figures S1, S2 and S3).

In the present case, *B. licheniformis* PL26 is able to produce both ε-polylysine and PHA in the same fermentation broth, which is not reported till date. However, *Streptomyces albulus* (CCRC 11814) is reported to produce ε-polylysine utilizing crude glycerol [3]. *S. albulus* being an Actinomycetes, it possesses a relatively slower growth rate as compared to *Bacillus* sp. As previously reported, *S. albulus* produces ε-polylysine after 120 h in M3G medium containing glucose as the carbon source, 0.2 g/L of ε-polylysine was produced by *Streptomyces albulus* (CCRC 11814) after 168 h [3], but in present study, ε-polylysine was produced after 96 h by *B. licheniformis* at a concentration of 0.2 g/L. In addition, fast growth rate and less production age of *B. licheniformis* which is producing 64.59% PHA with respect to dry cell weight i.e., 1.1 g/L P(3HB-co-3HV), which may be considered as the additional advantage of the process. Simultaneously, crude glycerol was replaced with analytical grade pure glycerol with similar concentration of the carbon source and it was found that pure glycerol yielded 0.06 g/L ε-polylysine and 0.4 g/L P(3HB-co-3HV).

0.2 g/L of ε-Poly-l-lysine produced from *Bacillus licheniformis* PL26 is in similar range with respect to its production from a wild strain *Streptomyces albulus* CCRC 11814 as reported in the literature [3]. However, similar sort of system developed by Moralejo-Ga’rate 2013, wherein using microbial community, simultaneous production of PHA and polyglucose was done [37]. Similarly, using halobacterium *Haloferax mediterranei*, simultaneously poly(3-hydroxybutyrate-*co*-3-hydroxyvalerate and extracellular polysaccharide (EPS) was produced [38]. Few microbes or microbial consortium have potential to produce two different polymers simultaneously using single carbon and nitrogen source.

### 3.2. Percentage Carbon Utilization of Bacillus licheniformis PL26

Carbon utilization percentage of isolated *Bacillus licheniformis* PL26 is shown in Table 1. *Bacillus licheniformis* isolated from salt pan has a 30% total carbon utilization percentage as it utilizes 0.21% total carbon from 0.7% total carbon present in the production medium.

### 3.3. Characterization of Purified PHA

The polymer extracted from *B. licheniformis* PL26 grown in production media was characterized through TGA, DSC, NMR and *Fourier transform infrared spectroscopy* (FTIR).

**Figure 1:**
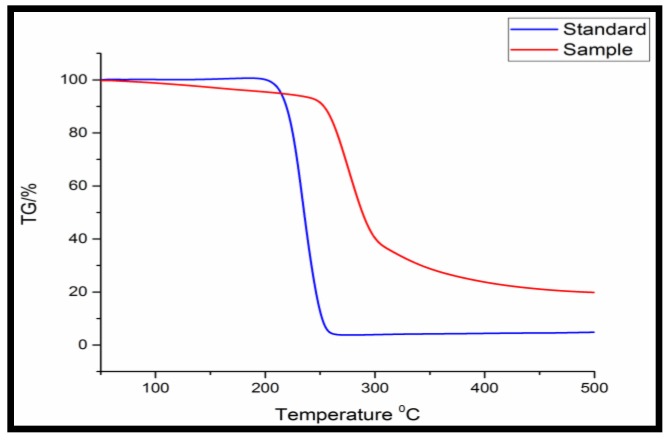
Thermogravimetric analysis (TGA) of purified PHA obtained from *Bacillus licheniformis*.

Figure 1 indicates TGA analysis to analyze the thermal decomposition of the extracted polymer through the thermogravimetric analyzer. The extracted polymer showed 0.28 g weight loss out of 0.35 g till 500 °C temperature. Mass change of 0.21 g PHA out of 0.35 g PHA was found in the temperature range of 225–325 °C.

**Figure 2:**
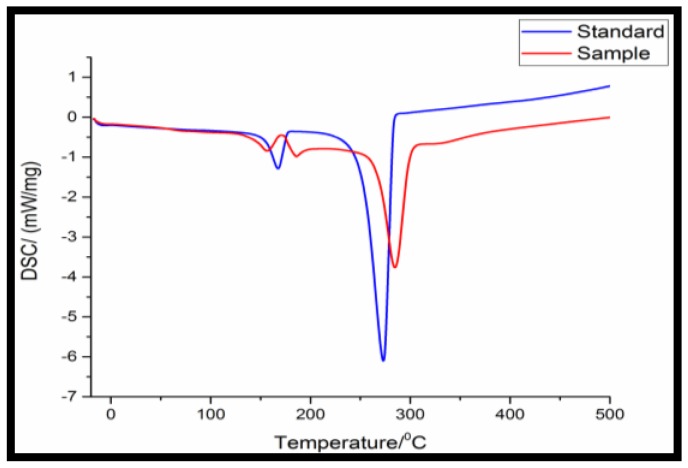
Differential scanning calorimetry (DSC) of purified PHA from *Bacillus licheniformis*.

The DSC analysis shown in Figure 2 indicates the melting temperature of the standard sample and the extracted polymer through sodium hypochlorite treatment. The thermal degradation was obtained at 268 °C for the obtained polymer and 250 °C of standard PHA from Sigma Aldrich.

**Figure 3:**
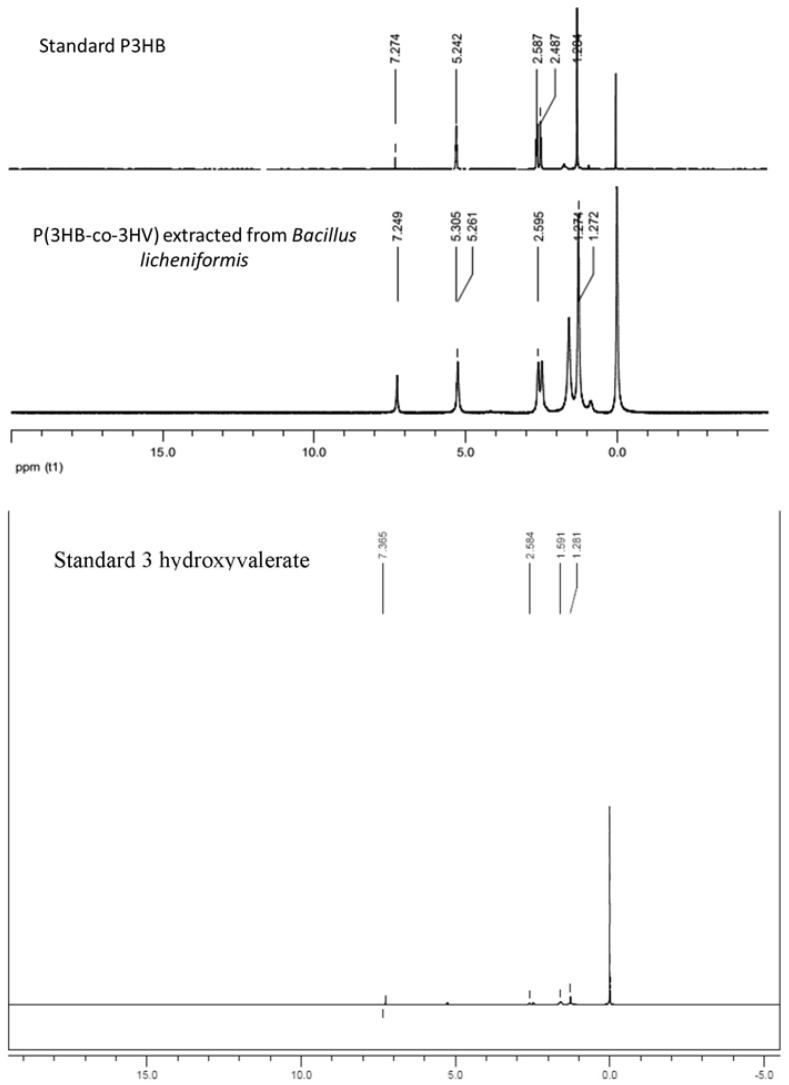
Nuclear magnetic resonance (NMR) spectra of purified product along with standard PHB and standard 3 hydroxy valerate.

^1^H NMR spectra of the extracted polymer, standard PHB and standard 3 hydroxy valerate were found to be comparable with respect to each other. Prominent peaks were observed at δ = 1.6 ppm for CH_3_, δ = 2.4 ppm for CH_2_ and δ = 5.2 ppm for CH group (Figure 3).

**Figure 4:**
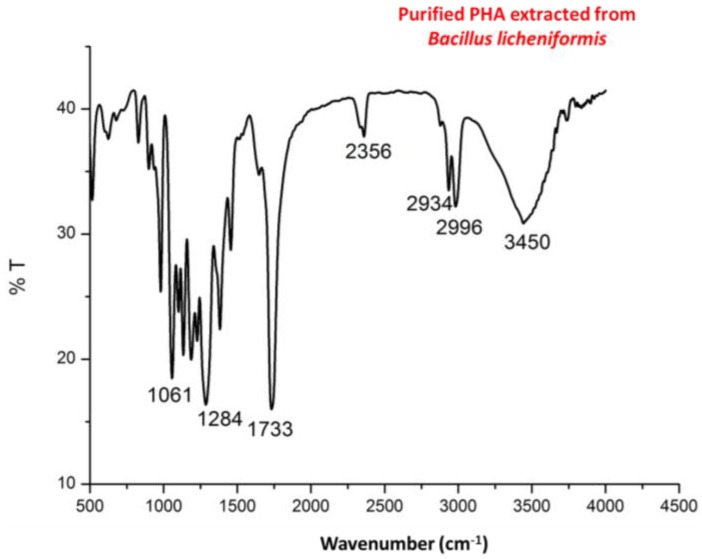
Fourier transform infrared spectroscopy (FTIR) spectra of purified PHA recovered from *Bacillus licheniformis*.

**Figure 5:**
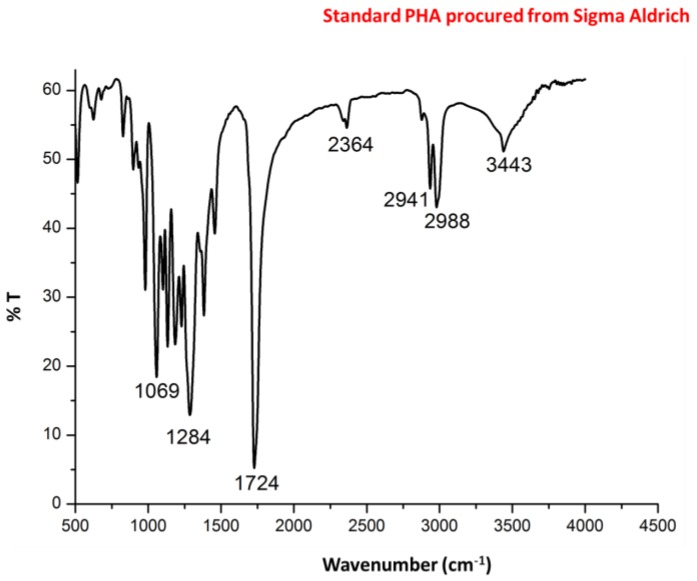
Fourier transform infrared spectroscopy (FTIR) spectra of standard PHA procured from Sigma Aldrich.

Infra red (IR) spectra (Figure 4) showed intense peaks at 1724 and 1283 cm^−1^ corresponding to –C=O and –CH group which are in correlation of peaks of standard PHA. Peaks at 1380, 1456 and 2932 correspond to –CH_3_, –CH_2_ and –CH group which are in correlation of peaks of standard PHA as shown in Figure 5.

### 3.4. Characterization of *ε*-Polylysine

#### ^1^H NMR of ε-Polylysine in D_2_O Isolated from *B. licheniformis* PL26

**Figure 6:**
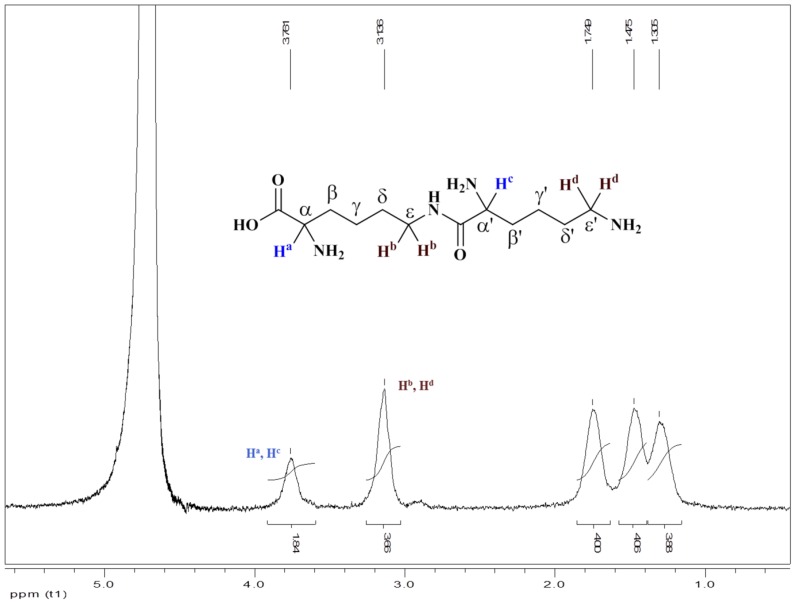
^1^H NMR of ε-PL in D_2_O isolated from *Bacillus licheniformis*.

Protons (H^a^, H^c^) attached to α-amino groups arrived together as broad singlet at δ 3.76 ppm and protons (H^b^, H^d^) attached to ε-amino groups arrived together as broad singlet at δ 3.14 ppm. Protons attached to β and β’ carbons come at δ 1.75 ppm as broad singlet. While the other protons attached to carbons come at δ 1.47 ppm (4H, ε and ε’) and δ 1.30 ppm (4H, γ and γ’ respectively (Figure 6). Overall, the peaks showing peptide linkage between α-carboxyl group and the ε-amino group, confirming the structure as ε-polylysine.

As per Jia et al., 2010, chemical shift of ε-H in the ε-polylysine units δ_εH_ and at the N-terminal δ’_εH_ are 3.097 and 2.863 of 5 KDa ε-polylysine protein [39]. However, similar results were obtained in the present case, wherein chemical shift of ε-H in the ε-polylysine units, δ_εH_ is at δ 3.14 ppm, which are in similar range with respect to the mentioned reports.

The described process showed the potential of utilizing *Bacillus licheniformis* for the production of PHA (64.59% w/w w.r.t cell dry mass i.e., 1.1 g/L P(3HB-co-3HV)) as well as ε-polylysine (0.2 g/L) using crude glycerol as the carbon source. In addition, there are no such reports as per our knowledge wherein two polymers are produced at a time in the same fermentation broth. Previously, Bera et al., 2014 reported microbial synthesis of such polymer (P(3HB-co-3HV)) by *Halomonas hydrothermalis* (MTCC accession no. 5445) from seaweed derived levulinic acid at a concentration of 57.5% PHA/dry cell weight and Ghosh et al., 2011 reported microbial synthesis of PHA from biodiesel by-products i.e., from crude glycerol and *Jatropha* deoiled cake hydrolysate at a concentration of 75% PHA/dry cell weight. However, further optimization will be required for efficient production of PHA and ε-polylysine at larger scale.

The most important parameter responsible for cost of the production in PHA is substrate for carbon and energy source. The economic feasibility of PHA would depend on few important factors like growth rate of microbe for generation of biomass, substrate cost and recovery process including the solvent involved. However, production of other biopolymer along with PHA in the fermentation medium will have additional advantage in further reducing the production cost. PHA production from biodiesel waste stream can reduce production cost and at the same time solves the problem of waste disposal. In order to increase the PHA yield, the concentration of carbon source and other nutrient source desired for the microbial production may be optimized.

## 4. Conclusions

In the present study, an integrated process for simultaneous production of extracellular ε-polylysine and intracellular P(3HB-co-3HV) developed through marine bacterial strain (*Bacillus licheniformis*) isolated from west coast of India utilizing Jatropha biodiesel waste residues as carbon rich source. A maximum of 0.2 g/L ε-polylysine content and 64.6% (w/w) P(3HB-co-3HV) production with respect to dry biomass was obtained in the fermentation broth using *Bacillus licheniformis*. ε-polylysine and PHA are important class of biopolymers which have various applications in food, agriculture, medicine, pharmacy, controlled drug release, tissue engineering, etc. Although, the present approach provides a solution for the effective utilization of biodiesel by-product, still, the developed process needs to be optimized further for gaining still better yield of both the products for its acclamation as cost effective and sustainable process.

## Figures and Tables

**Table 1:** Percentage carbon utilization by *Bacillus licheniformis.*

Parameter	Concentration
Total Carbon content in fermentation medium	0.7%
Total Carbon left in the supernatant after complete fermentation (96 h production age)	0.41%
Carbon present in the biomass	0.21%
Percentage carbon utilized	30%

## Supplementary Materials

The following are available online at http://www.mdpi.com/2306-5354/3/4/34/s1, Figure S1: effect of production age (h.) on ε-polylysine yield at 35 °C, Figure S2: effect of production age (h) on ε-polylysine yield at 37 °C, Figure S3: effect of production age (h) on ε-polylysine yield at 40 °C.
