# Supplementary material for: Biodegradable Polymeric Substances Produced by a Marine Bacterium from a Surplus Stream of the Biodiesel Industry
Source: Bioengineering (Basel). 2016 Nov 30;3(4):34. doi: 10.3390/bioengineering3040034 (PMC5597277; doi:10.3390/bioengineering3040034)
Supplement: Supplementary file 1 [file bioengineering-03-00034-s001.pdf]

bioengineering

Supplementary Materials: Biodegradable Polymeric Substances Produced by a Marine Bacterium from a Surplus Stream of the Biodiesel Industry

Sourish Bhattacharya, Sonam Dubey, Priyanka Singh, Anupama Shrivastava and Sandhya Mishra

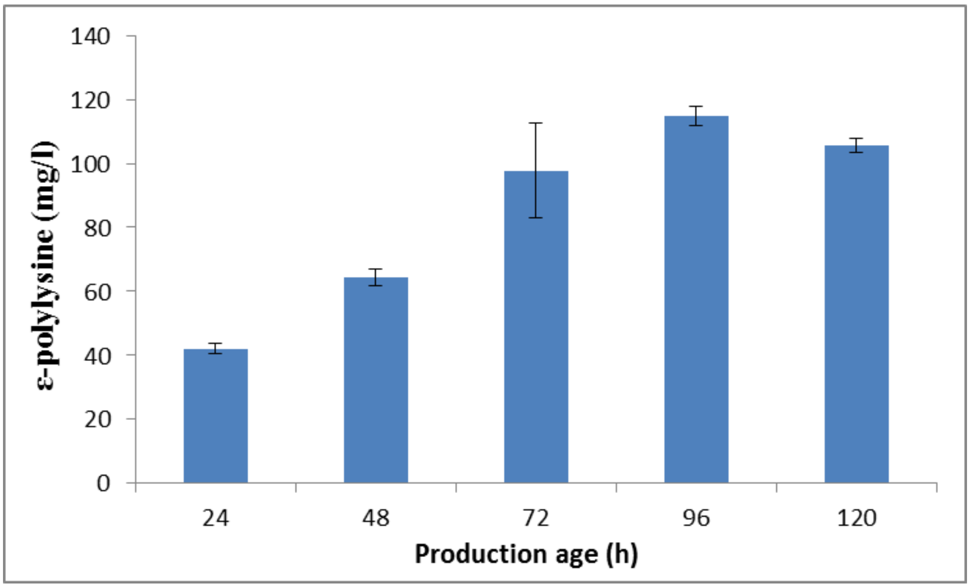

Figure S1. Effect of production age (h.) on ε-polylysine yield at 35 °C.

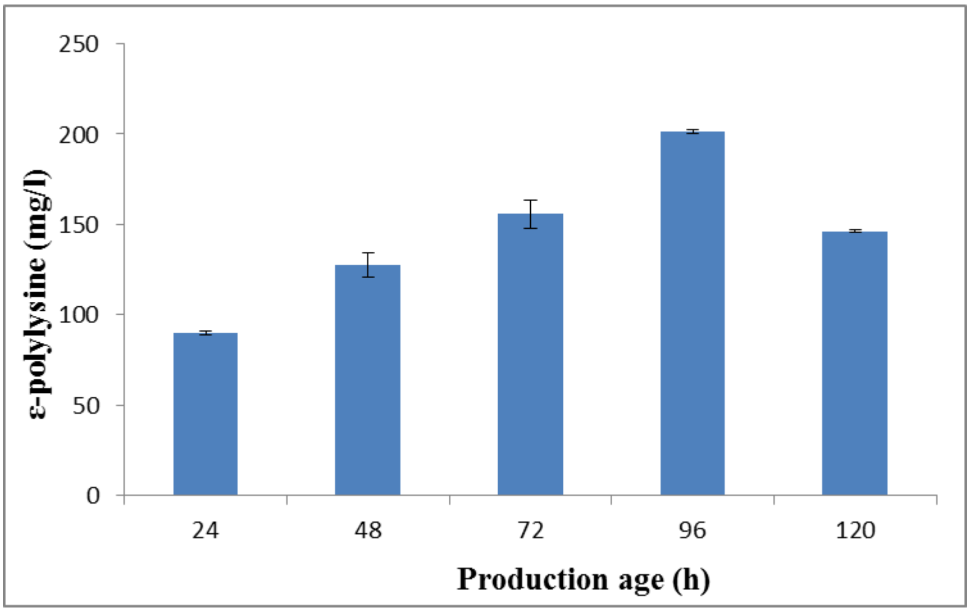

Figure S2. Effect of production age (h) on ε-polylysine yield at 37 °C.

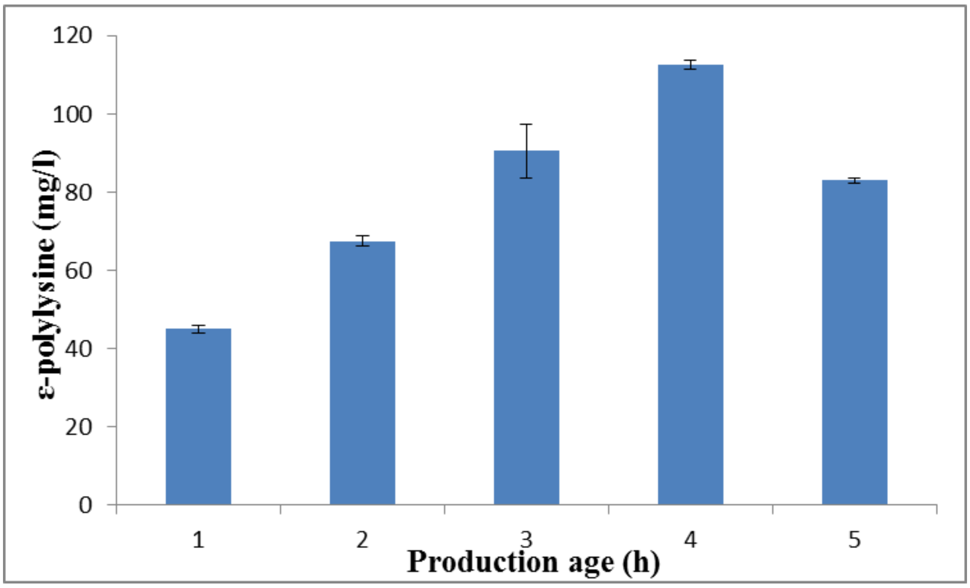

Figure S3. Effect of production age (h) on ε-polylysine yield at 40 °C.

© 2016 by the authors. Submitted for possible open access publication under the terms and conditions of the Creative Commons Attribution (CC-BY) license (http://creativecommons.org/licenses/by/4.0/).
